# 单中心移植后淋巴增殖性疾病的临床观察

**DOI:** 10.3760/cma.j.cn121090-20250319-00141

**Published:** 2025-12

**Authors:** 卓青 乔, 耀 孙, 艳萍 石, 博 彭, 婧 谢, 三春 蓝, 婧文 牛, 雷 王, 英杰 刘, 欲航 李, 江伟 扈, 娜 刘, 亮钉 胡

**Affiliations:** 中国人民解放军总医院第五医学中心血液病医学部，北京 100071 Department of Hematology, the Fifth Medical Center of Chinese People's Liberation Army General Hospital, Beijing 100071, China

## Abstract

为了分析异基因造血干细胞移植（allo-HSCT）后淋巴增殖性疾病的临床特征并探讨2022年世界卫生组织第五版《造血与淋巴组织肿瘤分类》（WHO-HAEM5）对移植后淋巴增殖性疾病（PTLD）的最新命名方式的临床指导意义，本文回顾性分析中国人民解放军总医院第五医学中心血液病医学部自2016年1月1日至2024年12月15日行allo-HSCT后诊断为PTLD的35例患者临床资料，其中临床诊断11例，病理确诊24例；24例病理确诊的PTLD按WHO-HAEM5最新命名方式重新命名。单形性PTLD，对应淋巴瘤亚型PTLD，该组患者预后最差，总生存率仅为54.5％（6/11），死亡患者原发病均为重型再生障碍性贫血（SAA），1例死于化疗相关并发症，其余4例均死于PTLD爆发式进展。原发病诊断为SAA的PTLD患者预后差，临床需采取更积极的干预策略。

## Abstract (en)

To analyze the clinical characteristics of post-allogeneic hematopoietic stem cell transplantation（allo-HSCT）lymphoproliferative disorders and to explore the clinical guidance provided by the latest nomenclature for post-transplant lymphoproliferative disorders（PTLD）in the 2022 World Health Organization Classification of Hematologic and Lymphoid Tumors（WHO-HAEM5）. A retrospective analysis was conducted on the clinical data of 35 patients diagnosed with PTLD after allo-HSCT at the Department of Hematology, Fifth Medical Center, Chinese PLA General Hospital, from January 1, 2016, to December 15, 2024. Among these, 11 cases were clinically diagnosed, and 24 cases were pathologically confirmed. The 24 pathologically confirmed PTLD cases were renamed according to the latest WHO-HAEM5 nomenclature. Monomorphic PTLD, corresponding to the lymphoma subtype of PTLD, exhibited the poorest prognosis with a over survival rate of only 54.5％（6/11）. All deceased patients had severe aplastic anemia（SAA）as the primary disease; one death resulted from chemotherapy-related complications, while the remaining four died due to explosive PTLD progression. PTLD patients with SAA as the primary diagnosis exhibit poor prognosis, necessitating more aggressive clinical intervention strategies.

随着新药或新的治疗方式的出现，一部分恶性血液病治疗模式发生改变，异基因造血干细胞移植（allo-HSCT）不再是唯一治愈手段，但其仍是适合移植的高危急性白血病、高危骨髓增生异常综合征（MDS）、部分复发难治淋巴瘤以及重型再生障碍性贫血（SAA）患者的最佳治疗选择。然而急性或慢性移植物抗宿主病（GVHD）、移植相关血栓性微血管病、移植后闭塞性细支气管炎、移植后淋巴增殖性疾病（PTLD）等移植相关并发症的发生会增加患者死亡率。因此如何进一步认识和管理移植相关并发症、提高移植技术等尤为重要。allo-HSCT后EB病毒（EBV）相关的PTLD（EBV-PTLD）报道的发生率为0.8％～11.9％，部分患者可爆发性进展，如得不到及时诊治，死亡率为60％～80％；而及时、合理地治疗PTLD，患者的远期生存率可接近60％[1]。本文回顾性分析我中心2016年1月1日至2024年12月15日诊治的PTLD患者临床特征、诊治情况、临床转归、影响总生存（OS）的危险因素以及探讨2022年世界卫生组织（WHO）第五版《造血与淋巴肿瘤分类》（WHO-HAEM5）对PTLD命名方式更新的临床指导意义。

## 病例与方法

1. 病例：本研究为回顾性研究，纳入中国人民解放军总医院第五医学中心2016年1月1日至2024年12月15日行allo-HSCT的患者，共701例次，共有35例患者诊断为PTLD。PTLD的诊断标准参考《造血干细胞移植后EB病毒相关淋巴增殖性疾病中国专家共识（2022年版）》[1]，分为临床诊断的PTLD及病理确诊的PTLD。进一步分析这些患者的移植相关特征及PTLD的相关特征及诊治情况，并对病理确诊病例按WHO-HAEM5重新命名。本研究已获得中国人民解放军总医院医学伦理委员会的批准（批件号：KY-2024-12-198-1）并免除签署患者知情同意书。

2. 移植预处理方案及GVHD预防方案：预处理方案：AML或MDS选用BU+CY（白消安+环磷酰胺）或FLU+BU（氟达拉滨+白消安）为基础的方案，ALL选用CY+TBI（环磷酰胺+全身照射）为基础的方案，SAA选用FC（氟达拉滨+环磷酰胺）±TBI的方案。GVHD预防选用：兔抗人胸腺细胞免疫球蛋白（rATG）、环孢素、短程甲氨蝶呤（MTX）、吗替麦考酚酯（MMF）±巴利昔单抗。需特别说明的是，同胞全相合移植，不用rATG，或用rATG 2.5 mg/kg×1 d；亲缘半相合或无关相合移植用rATG 2.5 mg/kg×3 d；SAA患者rATG加量为3.75 mg/kg×3 d或2.5 mg/kg×5 d；1例无关相合移植的AML患者使用后置环磷酰胺（PTCy）预防GVHD。

3. 移植后巨细胞病毒（CMV）及EBV监测：造血植入后开始监测CMV及EBV DNA定量，每周2次，持续至移植后3个月，合并GVHD患者或CMV和（或）EBV血症的患者，监测时间适当延长；疑诊PTLD患者，常规筛查CMV及EBV DNA定量。

4. 随访：通过查阅病历及电话联系进行随访，随访时间截至2025年2月16日。中位随访时间为17.87（0.36～109.17）个月。OS期定义为自PTLD诊断到死亡或最后一次随访的时间。

5. 统计学处理：数据分析采用SPSS 25.0软件进行。计量资料用*M*（范围）表示，计数资料用例数（百分率）表示。采用Kaplan-Meier法以及Log-rank检验，进行生存预后的单因素分析。基于临床专业知识和单因素分析结果（设定纳入标准为*P*<0.05），将有意义的变量纳入Cox多因素风险回归模型，以评估其对于生存预后的独立影响。采用向后逐步回归法（剔除标准为*P*>0.05）并结合似然比检验，以确定最终的独立预测因素。模型中的连续变量经检验满足比例风险假定。双侧*P*<0.05为差异有统计学意义。

## 结果

1. 一般资料：701例次移植，共诊断PTLD 35例，11例临床诊断，24例病理确诊，35例PTLD患者移植相关特征、诊治情况见表1。移植物选择，对于SAA，存在亲缘供者且同意捐献骨髓的患者采用骨髓（BM）联合或不联合外周血干细胞（PBSC）移植，其余均采用PBSC移植。PTLD临床表现特殊病例：1例以意识障碍为表现，无发热，无淋巴结肿大，EBV-DNA定量结果在2周内由低于检测下限快速升至1.942×10^5^ IU/ml，患者病情重，无法外出行头颅MRI或PET-CT检查，不排除PTLD相关脑病表现可能，予以供者照射淋巴细胞回输2次后，EBV转阴，临床症状好转；1例以严重的口腔溃疡为表现，行PET-CT可见溃疡深部为异常高代谢灶，最大标准摄取值为11.9，溃疡处口腔黏膜病理活检诊断为弥漫大B细胞淋巴瘤（DLBCL）。

**表1:** 35例移植后淋巴增殖性疾病（PTLD）患者移植相关特征、诊治情况［例（％）］

临床特征	统计值
性别	
男	26（74.29）
女	9（25.71）
移植时年龄［岁，*M*（范围）］	29（13~57）
原发病类型	
AML	11（31.43）
ALL	5（14.29）
MDS	2（5.71）
SAA	13（37.14）
其他^a^	4（11.43）
移植类型	
同胞全相合	8（22.86）
无关相合	7（20.00）
亲缘半相合	20（57.14）
预处理方案	
BU+CY	4（11.43）
CY+TBI	9（25.71）
FB	11（31.43）
FC+TBI	11（31.43）
造血干细胞来源	
外周血	28（80.00）
骨髓	1（2.86）
外周血+骨髓	6（17.14）
rATG用量（mg/kg）	
0^b^	3（8.57）
2.5	2（5.71）
7.5	19（54.29）
>7.5	11（31.43）
诊断PTLD时距移植的时间［d，*M*（范围）］	61（32～947）
临床表现	
仅发热	1（2.86）
仅淋巴结肿大	9（25.71）
发热并淋巴结肿大	23（65.71）
合并脑病	6（17.14）
病理确诊病例具体分型^c^	
增生	5（20.83）
增殖	8（33.33）
淋巴瘤	11（45.83）
PTLD治疗方案	
RIS	3（8.57）
RIS+细胞治疗	1（2.86）
R	22（62.86）
R+细胞治疗	4（11.43）
R-CHOP	4（11.43）
放弃治疗	1（2.86）

**注** AML：急性髓系白血病；ALL：急性淋巴细胞白血病；MDS：骨髓增生异常综合征；SAA：重型再生障碍性贫血；BU：白消安；CY：环磷酰胺；TBI：全身照射；FB：氟达拉滨+白消安；FC：氟达拉滨+环磷酰胺；rATG：兔抗人胸腺细胞免疫球蛋白；RIS：免疫抑制剂减量；R：利妥昔单抗；R-CHOP：利妥昔单抗+环磷酰胺+阿柔比星或多柔比星脂质体+长春新碱或长春地辛+泼尼松；^a^包括2例外周T细胞淋巴瘤非特指型、1例母细胞性浆细胞样树突状细胞肿瘤、1例多发性骨髓瘤；^b^其中包含1例使用后置环磷酰胺的患者；^c^共24例病理确诊

2. 诊断PTLD时CMV及EBV感染情况：①诊断PTLD时CMV感染情况：12例阳性，患者均为移植后120 d内发生的PTLD；②诊断PTLD时EBV感染情况：1例EBV-DNA定量低于检测下限，其余均为阳性，EBV-DNA中位值为4.823×10^4^ IU/ml（范围：5.460×10^2^～4.609×10^6^ IU/ml）；③移植后120 d内发生PTLD的患者有28例，其中12例EBV阳性的同时伴CMV阳性（包括1例应用来特莫韦预防，出现CMV突破感染且EBV阳性）；5例应用来特莫韦预防CMV血症，CMV阴性，EBV阳性；11例未用来特莫韦预防，CMV阴性而EBV阳性。

3. EBV感染细胞情况：共有3例患者进行流式细胞术分选后的EBV核酸定量。其中1例确诊PTLD时可见T细胞及B细胞均感染EBV，经利妥昔单抗单药治疗后3周复查流式细胞术分选的EBV核酸定量均转阴；1例可见T、B、NK细胞均感染EBV，且拷贝数较高（依次为5.87×10^4^拷贝数/ml、1.35×10^5^拷贝数/ml、1.55×10^5^拷贝数/ml），病理类型为EBV阳性DLBCL，虽然给予联合化疗，但患者病情迅速进展，合并脑病，最终因PTLD进展死亡；1例虽然可见T、B、NK细胞均感染EBV，但T及NK细胞EBV拷贝数较低（分别为1.47×10^3^拷贝数/ml、65.10拷贝数/ml），且病理类型为增生，仅通过免疫抑制剂减量便可达到治愈。

4. 确诊病例按WHO-HAEM5命名及结局：24例病理确诊病例，按WHO-HAEM5重新命名情况见表2。非结构破坏性PTLD，共5例，对应增生性病变亚型PTLD，其OS率为100％（5/5）；多形性PTLD，共8例，对应多形性淋巴细胞增殖性疾病，是具有不同恶性潜能的淋巴组织增殖性疾病，其OS率为62.5％（5/8），死亡的3例患者中，1例死于AML复发，1例原发病诊断SAA，死于PTLD进展，1例原发病诊断AML，死于化疗相关并发症；单形性PTLD，共11例，对应淋巴瘤亚型PTLD，患者预后较其他两组差，OS率只有54.5％（6/11），死亡患者均为SAA，1例死于化疗相关并发症，其余4例均死于PTLD爆发式进展。

**表2:** 24例移植后淋巴增殖性疾病（PTLD）病理确诊病例的世界卫生组织（WHO）第五版《造血与淋巴肿瘤分类》最新命名及治疗结局

例号	性别	年龄	诊断	移植类型	第4版命名	第5版命名	生存状态
1	男	41	AML	Haplo	非结构破坏性PTLD	浆细胞增生，EBV+，HSCT后	存活
2	女	53	AML	MUD	非结构破坏性PTLD	传染性单核细胞增多症样增生，EBV+，HSCT后	存活
3	男	24	MDS	Haplo	非结构破坏性PTLD	淋巴细胞增生伴组织细胞坏死，EBV+，HSCT后	存活
4	女	13	AML	Haplo	非结构破坏性PTLD	滤泡增生，EBV+，HSCT后	存活
5	男	17	SAA	MUD	非结构破坏性PTLD	滤泡增生，EBV+，HSCT后	存活
6	女	44	MDS	MSD	多形性PTLD	多形性淋巴细胞增殖性疾病，EBV−，HSCT后	存活
7	男	20	AML	Haplo	多形性PTLD	多形性淋巴细胞增殖性疾病，EBV+，HSCT后	存活
8	男	38	AML	MSD	多形性PTLD	多形性淋巴细胞增殖性疾病，EBV+，HSCT后	死亡
9	男	41	ALL	Haplo	多形性PTLD	多形性淋巴细胞增殖性疾病，EBV+，HSCT后	存活
10	女	26	ALL	Haplo	多形性PTLD	多形性淋巴细胞增殖性疾病，EBV+，HSCT后	存活
11	男	42	AML	Haplo	多形性PTLD	多形性淋巴细胞增殖性疾病，EBV+，HSCT后	死亡
12	男	20	SAA	MUD	多形性PTLD	多形性淋巴细胞增殖性疾病，EBV+，HSCT后	死亡
13	女	54	AML	MSD	多形性PTLD	多形性淋巴细胞增殖性疾病，EBV+，HSCT后	存活
14	男	55	AML	MUD	单形性PTLD	DLBCL，EBV+，HSCT后	存活
15	男	21	SAA	Haplo	单形性PTLD	PBL，EBV+，HSCT后	存活
16	男	24	BPDCN	Haplo	单形性PTLD	浆细胞瘤，EBV+，HSCT后	存活
17	男	33	SAA	MSD	单形性PTLD	DLBCL，EBV+，HSCT后	存活
18	男	21	SAA	MSD	单形性PTLD	DLBCL，EBV+，HSCT后	死亡
19	女	43	ALL	Haplo	单形性PTLD	B细胞淋巴瘤，EBV+，HSCT后	存活
20	男	24	SAA	MUD	单形性PTLD	DLBCL，EBV+，HSCT后	死亡
21	女	49	SAA	Haplo	单形性PTLD	PBL，EBV+，HSCT后	死亡
22	男	40	SAA	MSD	单形性PTLD	DLBCL，EBV+，HSCT后	死亡
23	男	26	ALL	Haplo	单形性PTLD	非霍奇金T细胞淋巴瘤，EBV+，HSCT后	存活
24	男	45	SAA	MSD	单形性PTLD	DLBCL，EBV+，HSCT后	死亡

**注** AML：急性髓系白血病；Haplo：亲缘半相合；EBV：EB病毒；HSCT：造血干细胞移植；MUD：无关相合；MDS：骨髓增生异常综合征；SAA：重型再生障碍性贫血；MSD：同胞全相合；ALL：急性淋巴细胞白血病；DLBCL：弥漫大B细胞淋巴瘤；PBL：浆母细胞淋巴瘤；BPDCN：母细胞性浆细胞样树突状细胞肿瘤

5. 生存分析：35例PTLD患者，24例（68.57％）存活，11例（31.43％）死亡，死亡的原因分别是：PTLD进展（6例，17.14％）、化疗相关并发症（4例，11.43％）、原发病复发（1例，2.86％）。Kaplan-Meier分析提示，良性淋巴组织增生、具有不同恶性潜能的多形性淋巴细胞增殖性疾病、恶性淋巴瘤这三类PTLD患者的生存差异无统计学意义（1年的OS率：100％对75.0％对54.5％，*P*＝0.243）。

采用Kaplan-Meier法进行生存预后的单因素分析，纳入性别、年龄是否大于40岁、原发病是否诊断为SAA、移植类型、预处理方案、rATG用量、诊断PTLD是否在移植后120 d内、是否应用来特莫韦预防CMV感染、病理类型因素，结果显示：原发病是否诊断SAA（*χ*^2^＝10.836，*P*＝0.001）与生存相关；进行Cox回归分析，结果显示，原发疾病为SAA是患者生存的独立危险因素（*HR*＝17.861，95％ *CI*：2.329～136.974，*P*＝0.006）。

## 讨论

PTLD是allo-HSCT后罕见但病死率较高的并发症[2]–[6]。本研究通过对单中心35例allo-HSCT后PTLD进行回顾性分析，系统评估了PTLD的临床病理特征及预后因素，并依据WHO-HAEM5对24例病理确诊病例重新诊断，结果支持了该分类系统的预后分层价值。

研究中我们发现根据WHO-HAEM5归类为淋巴瘤亚型的PTLD进展最快、预后最差。WHO-HAEM5将PTLD与其他免疫缺陷相关淋巴增殖性疾病统一归类，并采用“病理类型-病原学-免疫缺陷类型”的三部分命名法（如“DLBCL，EBV+，造血干细胞移植后”）[7]。该命名体系在凸显PTLD从良性至恶性的疾病谱系连续性的同时[6]，也有助于识别EBV阴性等少见亚型，揭示疾病的异质性。本研究的核心在于依据WHO-HAEM5对病理确诊的PTLD病例进行系统性再分类。结果显示，良性淋巴组织增生、具有不同恶性潜能的多形性淋巴细胞增殖性疾病、恶性淋巴瘤这三类PTLD患者的生存率呈递降趋势。这一发现不仅支持了新分类的理论合理性，也凸显其临床实用价值，有助于早期识别预后不良的淋巴瘤亚型，为实施更积极的干预提供病理依据。在防治方面，目前PTLD缺乏确切的预防策略[1]，抢先治疗在去T细胞allo-HSCT中尤为重要[8]。尽管启动治疗的EBV-DNA阈值尚未统一（通常在1 000～40 000拷贝数/ml）[9]–[10]，一项前瞻性研究显示，抢先使用利妥昔单抗可显著降低去T细胞移植患者PTLD发生率及相关死亡率（0％对26％）[11]。基于本研究的结果，淋巴瘤的亚型具有进展快、预后差的临床特点，提示在临床实践中需要采取更积极的干预策略。本研究中良、恶性组间生存率差异无统计学意义，可能与样本量少，死亡病例生存时间相对集中削弱组间差异有关。

本研究也观察到SAA较其他疾病，PTLD发生率、病理类型为淋巴瘤的概率更高且OS更差。这可能与SAA患者移植后应用更强的免疫抑制治疗、T细胞功能重建延迟有关[1],[12]–[13]。这提示对于原发疾病为SAA的单形性PTLD患者，需要采取更积极的临床策略，如EBV特异性细胞毒性T淋巴细胞（EBV-CTL）预防性输注[14]–[17]。

综上所述，移植后PTLD作为移植后死亡率极高的并发症，WHO-HAEM5对其的重新分类及命名，强调了疾病从良性增生至恶性改变的连续变化过程。本研究通过对PTLD的临床病理特征与预后分析，发现PTLD中被归类为淋巴瘤的亚型具有进展快、预后差的临床特点；此外，研究提示原发病为SAA的PTLD患者，其病理类型为淋巴瘤概率更高，且OS期更短。本研究为单中心回顾性设计，且样本量少，未来仍需大规模数据进一步验证。

## References

[1] 中华医学会血液学分会, 中国医师协会血液科医师分会 (2022). 造血干细胞移植后EB病毒相关淋巴增殖性疾病中国专家共识(2022年版)[J]. 中华血液学杂志.

[2] 顾 斌, 陈 广华, 王 荧 (2012). 异基因造血干细胞移植后淋巴细胞增殖性疾病的临床分析[J]. 中华血液学杂志.

[3] 许 兰平, 黄 晓军, 刘 代红 (2007). 造血干细胞移植后淋巴细胞增殖性疾病的临床研究[J]. 中华内科杂志.

[4] 牛 燕燕, 董 玉君, 尹 玥 (2021). 全血EB病毒DNA载量对异基因造血干细胞移植后淋巴增殖性疾病的诊断价值[J]. 中华血液学杂志.

[5] Choquet S (2024). Treatment of PTLD: a slow and difficult path[J]. Blood.

[6] El-Mallawany NK, Rouce RH (2024). EBV and post-transplant lymphoproliferative disorder: a complex relationship[J]. Hematology Am Soc Hematol Educ Program.

[7] Alaggio R, Amador C, Anagnostopoulos I (2022). The 5th edition of the World Health Organization Classification of Haematolymphoid Tumours: Lymphoid Neoplasms[J]. Leukemia.

[8] Clerico M, Dogliotti I, Aroldi A (2022). Post-transplant lymphoproliferative disease (PTLD) after allogeneic hematopoietic stem cell transplantation: biology and treatment options[J]. J Clin Med.

[9] 陈 静, 孙 于谦, 许 兰平 (2023). 血浆EB病毒DNA载量监测对单倍体造血干细胞移植后淋巴细胞增殖性疾病的预测价值[J]. 中华血液学杂志.

[10] Styczynski J, van der Velden W, Fox CP (2016). Management of Epstein-Barr Virus infections and post-transplant lymphoproliferative disorders in patients after allogeneic hematopoietic stem cell transplantation: Sixth European Conference on Infections in Leukemia (ECIL-6) guidelines[J]. Haematologica.

[11] van Esser JW, Niesters HG, van der Holt B (2002). Prevention of Epstein-Barr virus-lymphoproliferative disease by molecular monitoring and preemptive rituximab in high-risk patients after allogeneic stem cell transplantation[J]. Blood.

[12] 张 枫, 胡 冠华, 锁 盼 (2024). 利妥昔单抗为基础治疗儿童再生障碍性贫血单倍体移植后EB病毒相关淋巴细胞增殖性疾病的前瞻性单中心临床研究[J]. 中华血液学杂志.

[13] Lin F, Zhang Y, Han T (2022). A modified conditioning regimen based on low-dose cyclophosphamide and fludarabine for haploidentical hematopoietic stem cell transplant in severe aplastic anemia patients at risk of severe cardiotoxicity[J]. Clin Transplant.

[14] Atallah-Yunes SA, Salman O, Robertson MJ (2023). Post-transplant lymphoproliferative disorder: Update on treatment and novel therapies[J]. Br J Haematol.

[15] Al Hamed R, Bazarbachi AH, Mohty M (2020). Epstein-Barr virus-related post-transplant lymphoproliferative disease (EBV-PTLD) in the setting of allogeneic stem cell transplantation: a comprehensive review from pathogenesis to forthcoming treatment modalities[J]. Bone Marrow Transplant.

[16] Amengual JE, Pro B (2023). How I treat posttransplant lymphoproliferative disorder[J]. Blood.

[17] Aghsaeifard Z, Alizadeh R (2022). Clinical post-transplant lymphoproliferative disorders[J]. Cardiovasc Hematol Disord Drug Targets.

